# Supplementary material for: Robust inference in summary data Mendelian randomization via the zero modal pleiotropy assumption
Source: Int J Epidemiol. 2017 Jul 12;46(6):1985–98. doi: 10.1093/ije/dyx102 (PMC5837715; doi:10.1093/ije/dyx102)
Supplement: Supplementary Table S1 [file ije-2017-03-0276-file005_dyx102.docx]

**Supplementary Table 1. Mean estimates from simulation 1: directional horizontal pleiotropy under the InSIDE assumption and zero causal effect (10,000 simulations per scenario). In all cases,** $\boldsymbol{\varphi}$**=0.5.**

| **Estimator** | **Statistic** | **Proportion (%) of invalid instruments (Mean** $\frac{{\bar{\boldsymbol{F}}}_{\boldsymbol{GX}}\boldsymbol{-1}}{{\bar{\boldsymbol{F}}}_{\boldsymbol{GX}}}$ **[%]; mean** $\boldsymbol{I}_{\boldsymbol{GX}}^{\boldsymbol{2}}$ **[%])** | | | | | | | | | | |
| --- | --- | --- | --- | --- | --- | --- | --- | --- | --- | --- | --- | --- |
|  |  | 0 (99.7; 97.4) | 10 (99.7; 97.4) | 20 (99.7; 97.4) | 30 (99.7; 97.4) | 40 (99.7; 97.4) | 50 (99.7; 97.4) | 60 (99.7; 97.4) | 70 (99.7; 97.4) | 80 (99.7; 97.4) | 90 (99.7; 97.4) | 100 (99.7; 97.4) |
| Simple | Beta | 0.000 | 0.000 | 0.003 | 0.001 | 0.007 | 0.012 | 0.041 | 0.140 | 0.420 | 0.618 | 0.701 |
| Mode | SE | 0.058 | 0.067 | 0.068 | 0.076 | 0.086 | 0.111 | 0.111 | 0.149 | 0.207 | 0.206 | 0.213 |
|  | Coverage (%) | 99.6 | 99.5 | 99.4 | 99.2 | 98.8 | 97.9 | 92.5 | 69.5 | 30.7 | 11.8 | 7.4 |
|  | Power (%)^a^ | 0.4 | 0.5 | 0.6 | 0.8 | 1.2 | 2.1 | 7.5 | 30.5 | 69.3 | 88.2 | 92.6 |
| Weighted | Beta | 0.001 | 0.000 | 0.001 | 0.001 | 0.005 | 0.015 | 0.051 | 0.124 | 0.253 | 0.380 | 0.459 |
| Mode | SE | 0.052 | 0.060 | 0.061 | 0.069 | 0.078 | 0.102 | 0.099 | 0.109 | 0.140 | 0.144 | 0.156 |
|  | Coverage (%) | 99.8 | 99.4 | 99.3 | 98.7 | 97.7 | 94.9 | 81.8 | 48.7 | 25.0 | 12.4 | 7.6 |
|  | Power (%)^a^ | 0.2 | 0.6 | 0.7 | 1.4 | 2.3 | 5.1 | 18.2 | 51.3 | 75.0 | 87.6 | 92.4 |
| Simple | Beta | 0.000 | 0.000 | 0.003 | 0.001 | 0.007 | 0.012 | 0.041 | 0.140 | 0.420 | 0.618 | 0.701 |
| Mode | SE | 0.046 | 0.046 | 0.045 | 0.042 | 0.041 | 0.043 | 0.049 | 0.081 | 0.110 | 0.106 | 0.101 |
| (Under | Coverage (%) | 99.6 | 99.4 | 99.2 | 98.9 | 98.4 | 97.1 | 90.5 | 63.7 | 23.7 | 6.1 | 2.3 |
| NOME) | Power (%)^a^ | 0.4 | 0.6 | 0.8 | 1.1 | 1.6 | 2.9 | 9.5 | 36.3 | 76.3 | 93.9 | 97.7 |
| Weighted | Beta | 0.001 | 0.000 | 0.002 | 0.002 | 0.007 | 0.027 | 0.104 | 0.251 | 0.432 | 0.550 | 0.612 |
| Mode | SE | 0.040 | 0.039 | 0.038 | 0.036 | 0.035 | 0.038 | 0.045 | 0.058 | 0.067 | 0.066 | 0.067 |
| (Under | Coverage (%) | 99.8 | 99.4 | 99.1 | 98.1 | 97.0 | 92.2 | 71.6 | 31.2 | 10.5 | 3.6 | 1.8 |
| NOME) | Power (%)^a^ | 0.2 | 0.6 | 0.9 | 1.9 | 3.0 | 7.8 | 28.4 | 68.8 | 89.5 | 96.4 | 98.2 |

InSIDE: Instrument Strength Independent on Direct Effect. IVW: Inverse-variance weighting. SE: estimated standard error. NOME: NO Measurement Error.

^a^Given that the true causal effect is zero, power can be interpreted as the type-I error rate.
