# Supplementary material for: Robust inference in summary data Mendelian randomization via the zero modal pleiotropy assumption
Source: Int J Epidemiol. 2017 Jul 12;46(6):1985–98. doi: 10.1093/ije/dyx102 (PMC5837715; doi:10.1093/ije/dyx102)
Supplement: Supplementary Table S2 [file ije-2017-03-0276-file006_dyx102.docx]

**Supplementary Table 2. Mean estimates from simulation 2: inverse variance weighted^a^ Pearson correlation between instrument-exposure** ${\hat{\boldsymbol{\beta}}}_{\boldsymbol{X}}$ **and instrument-outcome associations** ${\hat{\boldsymbol{\beta}}}_{\boldsymbol{Y}}$**, and MR-Egger causal effect estimate** $\hat{\boldsymbol{\beta}}$**.**

| **Proportion of invalid instruments** | **Correlation between** ${\hat{\boldsymbol{\beta}}}_{\boldsymbol{X}}$ **and** ${\hat{\boldsymbol{\beta}}}_{\boldsymbol{Y}}$**^b^** | **MR-Egger** $\hat{\boldsymbol{\beta}}$ |
| --- | --- | --- |
| 0% | 0.002 | 0.001 |
| 10% | 0.298 | 0.111 |
| 20% | 0.409 | 0.188 |
| 30% | 0.469 | 0.240 |
| 40% | 0.504 | 0.274 |
| 50% | 0.529 | 0.294 |
| 60% | 0.538 | 0.303 |
| 70% | 0.541 | 0.302 |
| 80% | 0.533 | 0.288 |
| 90% | 0.515 | 0.263 |
| 100% | 0.486 | 0.223 |

^a^Implemented by dividing both $\hat{\beta}_{X}$ and $\hat{\beta}_{Y}$ by ${\sigma_{Y}}_{j}$ before computing the correlation.

^b^Since the true causal effect is zero, $\hat{\beta}_{Y}$ correspond to estimates of horizontal pleiotropic effects.
