# Supplementary material for: Robust inference in summary data Mendelian randomization via the zero modal pleiotropy assumption
Source: Int J Epidemiol. 2017 Jul 12;46(6):1985–98. doi: 10.1093/ije/dyx102 (PMC5837715; doi:10.1093/ije/dyx102)
Supplement: Supplementary Table S3 [file ije-2017-03-0276-file007_dyx102.docx]

**Supplementary Table 3. Mean estimates from simulation 2: directional horizontal pleiotropy mediated by a single confounder of the exposure-outcome association (so violating the InSIDE assumption) and zero causal effect (10,000 simulations per scenario). In all cases,** $\boldsymbol{\varphi}$**=0.5.**

| **Estimator** | **Statistic** | **Proportion (%) of invalid instruments (Mean** $\frac{{\bar{\boldsymbol{F}}}_{\boldsymbol{GX}}\boldsymbol{-1}}{{\bar{\boldsymbol{F}}}_{\boldsymbol{GX}}}$ **[%]; mean** $\boldsymbol{I}_{\boldsymbol{GX}}^{\boldsymbol{2}}$ **[%])** | | | | | | | | | | | |
| --- | --- | --- | --- | --- | --- | --- | --- | --- | --- | --- | --- | --- | --- |
|  |  | 0 (99.7; 97.4) | 10 (99.7; 97.6) | 20 (99.7; 97.8) | 30 (99.8; 98.0) | 40 (99.8; 98.1) | 50 (99.8; 98.2) | 60 (99.8; 98.2) | 70 (99.8; 98.2) | 80 (99.8; 98.2) | 90 (99.8; 98.1) | 100 (99.8; 98.0) |  |
| Simple | Beta | 0.000 | 0.001 | 0.002 | 0.006 | 0.014 | 0.041 | 0.116 | 0.219 | 0.288 | 0.317 | 0.324 |  |
| Mode | SE | 0.058 | 0.058 | 0.056 | 0.059 | 0.063 | 0.080 | 0.097 | 0.089 | 0.075 | 0.067 | 0.063 |  |
|  | Coverage (%) | 99.6 | 99.6 | 99.5 | 99.2 | 98.1 | 92.4 | 71.2 | 38.9 | 16.9 | 7.3 | 4.9 |  |
|  | Power (%)^a^ | 0.4 | 0.4 | 0.5 | 0.8 | 1.9 | 7.7 | 28.8 | 61.1 | 83.1 | 92.7 | 95.1 |  |
| Weighted | Beta | 0.001 | 0.003 | 0.013 | 0.043 | 0.102 | 0.186 | 0.253 | 0.290 | 0.304 | 0.311 | 0.315 |  |
| Mode | SE | 0.052 | 0.052 | 0.054 | 0.063 | 0.065 | 0.061 | 0.055 | 0.050 | 0.045 | 0.044 | 0.042 |  |
|  | Coverage (%) | 99.8 | 99.4 | 95.9 | 86.7 | 68.2 | 41.5 | 20.2 | 8.6 | 4.0 | 2.2 | 1.6 |  |
|  | Power (%)^a^ | 0.2 | 0.6 | 4.1 | 13.3 | 31.8 | 58.5 | 79.8 | 91.4 | 96.0 | 97.8 | 98.4 |  |
| Simple | Beta | 0.000 | 0.001 | 0.002 | 0.006 | 0.014 | 0.041 | 0.116 | 0.219 | 0.288 | 0.317 | 0.324 |  |
| Mode | SE | 0.046 | 0.046 | 0.046 | 0.048 | 0.054 | 0.074 | 0.091 | 0.083 | 0.071 | 0.064 | 0.061 |  |
| (Under | Coverage (%) | 99.6 | 99.6 | 99.5 | 99.1 | 98.2 | 92.0 | 70.7 | 38.4 | 16.4 | 7.1 | 4.8 |  |
| NOME) | Power (%)^a^ | 0.4 | 0.4 | 0.5 | 0.9 | 1.8 | 8.0 | 29.3 | 61.6 | 83.6 | 92.9 | 95.2 |  |
| Weighted | Beta | 0.001 | 0.003 | 0.018 | 0.061 | 0.131 | 0.218 | 0.277 | 0.307 | 0.317 | 0.321 | 0.325 |  |
| Mode | SE | 0.040 | 0.040 | 0.046 | 0.054 | 0.057 | 0.052 | 0.047 | 0.043 | 0.041 | 0.040 | 0.040 |  |
| (Under | Coverage (%) | 99.8 | 99.0 | 94.6 | 81.2 | 59.5 | 32.9 | 14.4 | 5.5 | 2.8 | 1.4 | 1.1 |  |
| NOME) | Power (%)^a^ | 0.2 | 1.0 | 5.4 | 18.8 | 40.5 | 67.1 | 85.7 | 94.5 | 97.2 | 98.6 | 98.9 |  |

InSIDE: Instrument Strength Independent on Direct Effect. IVW: Inverse-variance weighting. SE: estimated standard error. NOME: NO Measurement Error.

^a^Given that the true causal effect is zero, power can be interpreted as the type-I error rate.
