# Supplementary material for: Robust inference in summary data Mendelian randomization via the zero modal pleiotropy assumption
Source: Int J Epidemiol. 2017 Jul 12;46(6):1985–98. doi: 10.1093/ije/dyx102 (PMC5837715; doi:10.1093/ije/dyx102)
Supplement: Supplementary Table S4 [file ije-2017-03-0276-file008_dyx102.docx]

**Supplementary Table 4. Mean estimates from simulation 3: no horizontal pleiotropy and causal effect** $\boldsymbol{\beta}$**=0.1 (10,000 simulations per scenario). Sample sizes** $\boldsymbol{N}_{\boldsymbol{X}}$ **and** $\boldsymbol{N}_{\boldsymbol{Y}}$ **are in thousands. In all cases,** $\boldsymbol{\varphi}$**=0.5.**

| **Estimator** | **Statistic** | **N** | **Mean** $\frac{{\bar{\boldsymbol{F}}}_{\boldsymbol{GX}}\boldsymbol{-1}}{{\bar{\boldsymbol{F}}}_{\boldsymbol{GX}}}$ **[%]; mean** $\boldsymbol{I}_{\boldsymbol{GX}}^{\boldsymbol{2}}$ **[%]** | | | | | | | | |
| --- | --- | --- | --- | --- | --- | --- | --- | --- | --- | --- | --- |
|  |  |  | 99.3; 94.8 | 99.3; 94.8 | 99.3; 94.8 | 99.7; 97.4 | 99.7; 97.4 | 99.7; 97.4 | 99.8; 98.7 | 99.8; 98.7 | 99.8; 98.7 |
|  |  | $\boldsymbol{N}_{\boldsymbol{X}}$ | 25 | 25 | 25 | 50 | 50 | 50 | 100 | 100 | 100 |
|  |  | $\boldsymbol{N}_{\boldsymbol{Y}}$ | 25 | 50 | 100 | 25 | 50 | 100 | 25 | 50 | 100 |
| Simple | Beta |  | 0.099 | 0.098 | 0.099 | 0.099 | 0.099 | 0.100 | 0.100 | 0.100 | 0.099 |
| Mode | SE |  | 0.102 | 0.072 | 0.055 | 0.091 | 0.058 | 0.044 | 0.072 | 0.051 | 0.036 |
|  | Coverage (%) |  | 99.4 | 99.4 | 99.5 | 99.7 | 99.6 | 99.5 | 99.6 | 99.5 | 99.7 |
|  | Power (%) |  | 29.1 | 58.3 | 85.3 | 30.4 | 61.2 | 88.7 | 31.7 | 63.1 | 90.1 |
| Weighted | Beta |  | 0.097 | 0.096 | 0.097 | 0.098 | 0.098 | 0.098 | 0.099 | 0.098 | 0.099 |
| Mode | SE |  | 0.093 | 0.065 | 0.050 | 0.082 | 0.052 | 0.039 | 0.063 | 0.044 | 0.032 |
|  | Coverage (%) |  | 99.6 | 99.6 | 99.7 | 99.7 | 99.8 | 99.8 | 99.6 | 99.6 | 99.7 |
|  | Power (%) |  | 41.2 | 69.8 | 90.2 | 43.2 | 73.9 | 93.4 | 44.5 | 76.0 | 95.2 |
| Simple | Beta |  | 0.099 | 0.098 | 0.099 | 0.099 | 0.099 | 0.100 | 0.100 | 0.100 | 0.099 |
| Mode | SE |  | 0.066 | 0.047 | 0.033 | 0.066 | 0.047 | 0.033 | 0.065 | 0.047 | 0.033 |
| (Under | Coverage (%) |  | 99.4 | 99.5 | 99.5 | 99.7 | 99.6 | 99.5 | 99.6 | 99.4 | 99.7 |
| NOME) | Power (%) |  | 31.5 | 62.3 | 89.6 | 31.9 | 63.2 | 91.2 | 32.3 | 63.9 | 91.0 |
| Weighted | Beta |  | 0.099 | 0.097 | 0.098 | 0.099 | 0.099 | 0.099 | 0.099 | 0.099 | 0.100 |
| Mode | SE |  | 0.057 | 0.040 | 0.029 | 0.057 | 0.040 | 0.028 | 0.056 | 0.040 | 0.028 |
| (Under | Coverage (%) |  | 99.5 | 99.6 | 99.6 | 99.6 | 99.7 | 99.7 | 99.7 | 99.6 | 99.7 |
| NOME) | Power (%) |  | 45.4 | 75.5 | 94.9 | 45.2 | 76.8 | 95.7 | 45.2 | 77.1 | 96.2 |

$N_{X}$: sample size of the dataset used to estimate instrument-exposure associations.$N_{Y}$: sample size of the dataset used to estimate instrument-outcome associations. IVW: Inverse-variance weighting. SE: estimated standard error. NOME: NO Measurement Error.
