# Supplementary material for: Robust inference in summary data Mendelian randomization via the zero modal pleiotropy assumption
Source: Int J Epidemiol. 2017 Jul 12;46(6):1985–98. doi: 10.1093/ije/dyx102 (PMC5837715; doi:10.1093/ije/dyx102)
Supplement: Supplementary Table S5 [file ije-2017-03-0276-file009_dyx102.docx]

**Supplementary Table 5. Mean estimates from simulation 4: no horizontal pleiotropy, zero causal effect and partially (50%) of fully (100%) overlapping samples (10,000 simulations per scenario). In all cases,** $\boldsymbol{\varphi}$**=1.**

| **Estimator** | **Statistic** | **N** | **Mean** $\frac{{\bar{\boldsymbol{F}}}_{\boldsymbol{GX}}\boldsymbol{-1}}{{\bar{\boldsymbol{F}}}_{\boldsymbol{GX}}}$ **[%]; mean** $\boldsymbol{I}_{\boldsymbol{GX}}^{\boldsymbol{2}}$ **[%]** | | | | | |
| --- | --- | --- | --- | --- | --- | --- | --- | --- |
|  |  |  | 89.2; 21.8 | 97.0; 77.0 | 98.4; 87.6 | 89.3; 22.0 | 97.0; 77.0 | 98.4; 87.7 |
|  |  |  | Sample overlap=50% | | | Sample overlap=100% | | |
|  |  | $\boldsymbol{N}_{\boldsymbol{X}}$ | 1,000 | 5,000 | 10,000 | 1,000 | 5,000 | 10,000 |
|  |  | $\boldsymbol{N}_{\boldsymbol{Y}}$ | 1,000 | 5,000 | 10,000 | 1,000 | 5,000 | 10,000 |
| IVW | Beta |  | 0.033 | 0.008 | 0.004 | 0.066 | 0.016 | 0.008 |
|  | SE |  | 0.093 | 0.046 | 0.033 | 0.093 | 0.046 | 0.033 |
|  | Coverage (%) |  | 94.9 | 96.3 | 96.2 | 90.6 | 95.2 | 95.7 |
|  | Power (%) |  | 5.1 | 3.7 | 3.8 | 9.4 | 4.8 | 4.3 |
| MR-Egger | Beta |  | 0.055 | 0.020 | 0.012 | 0.109 | 0.041 | 0.024 |
|  | SE |  | 0.176 | 0.096 | 0.070 | 0.176 | 0.095 | 0.070 |
|  | Coverage (%) |  | 95.6 | 96.2 | 96.1 | 92.4 | 94.6 | 95.2 |
|  | Power (%) |  | 4.4 | 3.8 | 3.9 | 7.6 | 5.4 | 4.8 |
| Weighted | Beta |  | 0.039 | 0.010 | 0.005 | 0.078 | 0.022 | 0.011 |
| Median | SE |  | 0.131 | 0.063 | 0.045 | 0.130 | 0.063 | 0.045 |
|  | Coverage (%) |  | 96.8 | 97.2 | 97.3 | 94.0 | 96.5 | 96.7 |
|  | Power (%) |  | 3.2 | 2.8 | 2.7 | 6.0 | 3.5 | 3.3 |
| Simple | Beta |  | 0.035 | 0.003 | 0.003 | 0.068 | 0.013 | 0.006 |
| Mode^b^ | SE |  | 2.001 | 0.403 | 0.209 | 1.967 | 0.362 | 0.209 |
|  | Coverage (%) |  | 99.1 | 99.1 | 99.1 | 98.8 | 99.1 | 99.2 |
|  | Power (%)^a^ |  | 0.9 | 0.9 | 0.9 | 1.2 | 0.9 | 0.8 |
| Weighted | Beta |  | 0.041 | 0.007 | 0.005 | 0.076 | 0.022 | 0.010 |
| Mode^b^ | SE |  | 1.969 | 0.387 | 0.197 | 1.936 | 0.345 | 0.197 |
|  | Coverage (%) |  | 99.3 | 98.7 | 98.6 | 98.2 | 98.2 | 98.3 |
|  | Power (%)^a^ |  | 0.7 | 1.4 | 1.4 | 1.8 | 1.8 | 1.7 |
| Simple | Beta |  | 0.035 | 0.003 | 0.003 | 0.068 | 0.013 | 0.006 |
| Mode | SE |  | 0.213 | 0.105 | 0.074 | 0.212 | 0.104 | 0.073 |
| (Under | Coverage (%) |  | 98.4 | 98.9 | 99.0 | 97.8 | 98.8 | 99.0 |
| NOME)^b^ | Power (%)^a^ |  | 1.6 | 1.1 | 1.0 | 2.2 | 1.3 | 1.0 |
| Weighted | Beta |  | 0.046 | 0.007 | 0.005 | 0.087 | 0.023 | 0.010 |
| Mode | SE |  | 0.168 | 0.086 | 0.060 | 0.167 | 0.085 | 0.060 |
| (Under | Coverage (%) |  | 96.8 | 97.7 | 97.9 | 93.6 | 96.9 | 97.6 |
| NOME)^b^ | Power (%)^a^ |  | 3.2 | 2.3 | 2.1 | 6.4 | 3.1 | 2.4 |

$N_{X}$: sample size of the dataset used to estimate instrument-exposure associations.$N_{Y}$: sample size of the dataset used to estimate instrument-outcome associations. IVW: Inverse-variance weighting. SE: estimated standard error. NOME: NO Measurement Error.

^a^Given that the true causal effect is zero, power can be interpreted as the type-I error rate.
