# Supplementary material for: Robust inference in summary data Mendelian randomization via the zero modal pleiotropy assumption
Source: Int J Epidemiol. 2017 Jul 12;46(6):1985–98. doi: 10.1093/ije/dyx102 (PMC5837715; doi:10.1093/ije/dyx102)
Supplement: Supplementary Table S6 [file ije-2017-03-0276-file010_dyx102.docx]

**Supplementary Table 6. Mean estimates from simulation 4: no horizontal pleiotropy, zero causal effect and partially (50%) of fully (100%) overlapping samples (10,000 simulations per scenario). In all cases,** $\boldsymbol{\varphi}$**=0.5.**

| **Estimator** | **Statistic** | **N** | **Mean** $\frac{{\bar{\boldsymbol{F}}}_{\boldsymbol{GX}}\boldsymbol{-1}}{{\bar{\boldsymbol{F}}}_{\boldsymbol{GX}}}$ **[%]; mean** $\boldsymbol{I}_{\boldsymbol{GX}}^{\boldsymbol{2}}$ **[%]** | | | | | |
| --- | --- | --- | --- | --- | --- | --- | --- | --- |
|  |  |  | 89.2; 21.8 | 97.0; 77.0 | 98.4; 87.6 | 89.3; 22.0 | 97.0; 77.0 | 98.4; 87.7 |
|  |  |  | Sample overlap=50% | | | Sample overlap=100% | | |
|  |  | $\boldsymbol{N}_{\boldsymbol{X}}$ | 1,000 | 5,000 | 10,000 | 1,000 | 5,000 | 10,000 |
|  |  | $\boldsymbol{N}_{\boldsymbol{Y}}$ | 1,000 | 5,000 | 10,000 | 1,000 | 5,000 | 10,000 |
| Simple | Beta |  | 0.032 | 0.004 | 0.002 | 0.072 | 0.015 | 0.008 |
| Mode | SE |  | 2.006 | 0.423 | 0.228 | 1.975 | 0.381 | 0.227 |
|  | Coverage (%) |  | 99.2 | 99.5 | 99.6 | 99.1 | 99.5 | 99.6 |
|  | Power (%)^a^ |  | 0.8 | 0.5 | 0.4 | 0.9 | 0.5 | 0.4 |
| Weighted | Beta |  | 0.037 | 0.008 | 0.005 | 0.079 | 0.024 | 0.013 |
| Mode | SE |  | 1.966 | 0.403 | 0.214 | 1.936 | 0.362 | 0.213 |
|  | Coverage (%) |  | 99.8 | 99.7 | 99.8 | 99.5 | 99.6 | 99.7 |
|  | Power (%)^a^ |  | 0.2 | 0.3 | 0.2 | 0.6 | 0.4 | 0.4 |
| Simple | Beta |  | 0.032 | 0.004 | 0.002 | 0.072 | 0.015 | 0.008 |
| Mode | SE |  | 0.292 | 0.147 | 0.104 | 0.290 | 0.146 | 0.103 |
| (Under | Coverage (%) |  | 99.2 | 99.6 | 99.6 | 99.0 | 99.5 | 99.6 |
| NOME) | Power (%)^a^ |  | 0.8 | 0.5 | 0.4 | 1.0 | 0.5 | 0.4 |
| Weighted | Beta |  | 0.042 | 0.009 | 0.005 | 0.090 | 0.025 | 0.013 |
| Mode | SE |  | 0.237 | 0.124 | 0.089 | 0.235 | 0.123 | 0.088 |
| (Under | Coverage (%) |  | 98.9 | 99.4 | 99.7 | 97.9 | 99.2 | 99.6 |
| NOME) | Power (%)^a^ |  | 1.1 | 0.6 | 0.3 | 2.1 | 0.8 | 0.4 |

$N_{X}$: sample size of the dataset used to estimate instrument-exposure associations.$N_{Y}$: sample size of the dataset used to estimate instrument-outcome associations. IVW: Inverse-variance weighting. SE: estimated standard error. NOME: NO Measurement Error.

^a^Given that the true causal effect is zero, power can be interpreted as the type-I error rate.
