# Supplementary material for: Robust inference in summary data Mendelian randomization via the zero modal pleiotropy assumption
Source: Int J Epidemiol. 2017 Jul 12;46(6):1985–98. doi: 10.1093/ije/dyx102 (PMC5837715; doi:10.1093/ije/dyx102)
Supplement: Supplementary Table S7 [file ije-2017-03-0276-file011_dyx102.docx]

**Supplementary Table 7.** Mean estimates from simulations 1 and 2 (zero causal effect; 10,000 simulations per scenario) of coverage of 95% confidence intervals of the mode estimate ($\varphi$=1) computed using bootstrap.

| **Method to compute** | **Proportion (%) of invalid instruments** | | | | | | | | | | |
| --- | --- | --- | --- | --- | --- | --- | --- | --- | --- | --- | --- |
| **confidence intervals** | **0** | **10** | **20** | **30** | **40** | **50** | **60** | **70** | **80** | **90** | **100** |
| ***Simulation 1: horizontal pleiotropy (InSIDE holds)*** | | | | | | | | | | | |
| **Simple mode** |  |  |  |  |  |  |  |  |  |  |  |
| Normal approximation | 99.2 | 98.8 | 98.5 | 97.9 | 96.8 | 87.0 | 37.4 | 9.9 | 5.6 | 4.4 | 4.1 |
| Percentile | 100.0 | 100.0 | 99.9 | 99.5 | 96.8 | 74.5 | 16.4 | 5.8 | 4.7 | 3.9 | 4.0 |
| **Weighted mode** |  |  |  |  |  |  |  |  |  |  |  |
| Normal approximation | 98.5 | 98.0 | 97.6 | 96.6 | 93.8 | 71.1 | 19.5 | 8.2 | 6.8 | 5.6 | 5.1 |
| Percentile | 99.7 | 99.4 | 98.7 | 97.0 | 89.9 | 57.0 | 15.8 | 8.0 | 7.0 | 6.0 | 5.6 |
| **Simple mode (under NOME)** |  |  |  |  |  |  |  |  |  |  |  |
| Normal approximation | 99.1 | 98.7 | 98.1 | 97.4 | 95.8 | 84.7 | 29.8 | 4.1 | 1.4 | 0.6 | 0.6 |
| Percentile | 100.0 | 99.9 | 99.7 | 99.1 | 95.9 | 69.8 | 9.1 | 0.8 | 0.5 | 0.3 | 0.3 |
| **Weighted mode (under NOME)** |  |  |  |  |  |  |  |  |  |  |  |
| Normal approximation | 98.3 | 97.6 | 97.2 | 95.8 | 92.3 | 65.8 | 12.0 | 2.3 | 1.2 | 0.7 | 0.8 |
| Percentile | 99.6 | 99.3 | 98.5 | 96.5 | 87.0 | 44.7 | 4.8 | 0.8 | 0.6 | 0.4 | 0.4 |
| ***Simulation 2: horizontal pleiotropy (InSIDE violated)*** | | | | | | | | | | | |
| **Simple mode** |  |  |  |  |  |  |  |  |  |  |  |
| Normal approximation | 99.1 | 98.8 | 98.6 | 98.2 | 96.5 | 86.2 | 56.4 | 21.6 | 4.9 | 1.7 | 0.6 |
| Percentile | 100.0 | 100.0 | 99.7 | 98.3 | 88.8 | 55.2 | 15.6 | 1.7 | 0.3 | 0.3 | 0.1 |
| **Weighted mode** |  |  |  |  |  |  |  |  |  |  |  |
| Normal approximation | 98.4 | 98.3 | 96.6 | 87.7 | 63.1 | 32.2 | 11.1 | 3.2 | 0.8 | 0.6 | 0.2 |
| Percentile | 99.8 | 99.1 | 96.2 | 79.8 | 44.3 | 12.6 | 2.2 | 0.9 | 0.3 | 0.3 | 0.1 |
| **Simple mode (under NOME)** |  |  |  |  |  |  |  |  |  |  |  |
| Normal approximation | 99.1 | 98.7 | 98.4 | 98.3 | 96.3 | 86.0 | 55.7 | 20.7 | 4.6 | 1.5 | 0.5 |
| Percentile | 100.0 | 99.9 | 99.6 | 98.1 | 88.3 | 53.9 | 14.8 | 0.9 | 0.0 | 0.0 | 0.0 |
| **Weighted mode (under NOME)** |  |  |  |  |  |  |  |  |  |  |  |
| Normal approximation | 98.1 | 98.0 | 96.1 | 83.7 | 56.1 | 24.8 | 7.5 | 1.6 | 0.5 | 0.1 | 0.1 |
| Percentile | 99.7 | 99.0 | 95.4 | 75.8 | 37.6 | 8.5 | 0.8 | 0.1 | 0.0 | 0.0 | 0.0 |

InSIDE: Instrument Strength Independent on Direct Effect. NOME: NO Measurement Error.
