# Supplementary figures and images for: Robust inference in summary data Mendelian randomization via the zero modal pleiotropy assumption
Source: Int J Epidemiol. 2017 Jul 12;46(6):1985–98. doi: 10.1093/ije/dyx102 (PMC5837715; doi:10.1093/ije/dyx102)

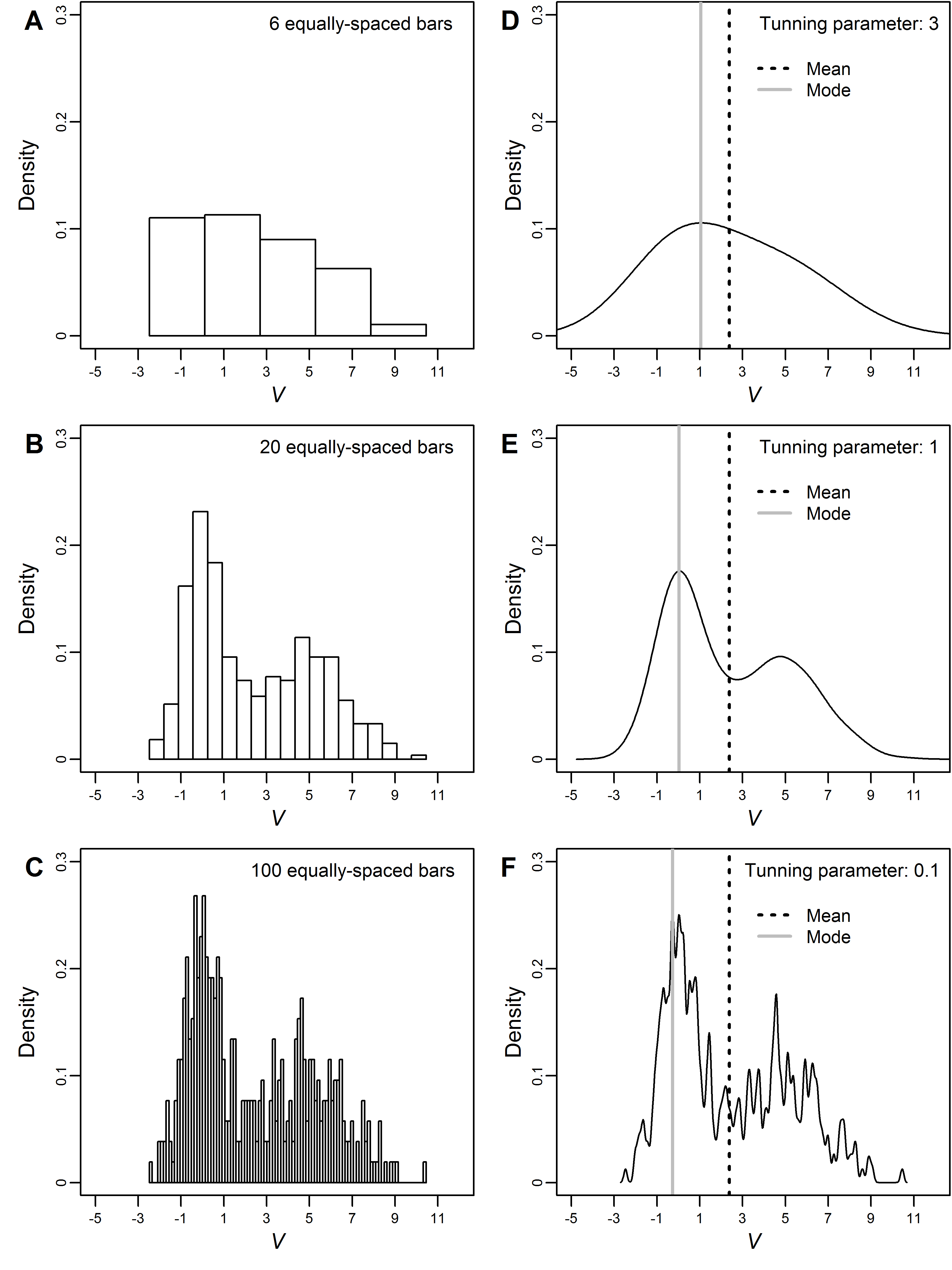

Supplement: Supplementary Figure S1 [file ije-2017-03-0276-file012_dyx102.png]
