# Supplementary material for: Robust inference in summary data Mendelian randomization via the zero modal pleiotropy assumption
Source: Int J Epidemiol. 2017 Jul 12;46(6):1985–98. doi: 10.1093/ije/dyx102 (PMC5837715; doi:10.1093/ije/dyx102)
Supplement: Supplementary Methods [file ije-2017-03-0276-file004_dyx102.docx]

**Supplementary Methods**

**Summary data Mendelian randomisation**

The IVW estimate for the causal effect is an inverse-variance weighted average of all ${\hat{\beta}_{R}}_{j}$s. Under models (1) and (2), variant $j$ is a valid instrument when $\alpha_{j}$=0 and invalid when $\alpha_{j}$≠0. When variant $j$ is a valid instrument, ${\hat{\beta}_{R}}_{j}$ is a consistent estimate for the true causal effect $\beta$ (i.e., ${\hat{\beta}_{R}}_{j}$tends to ${\beta_{R}}_{j}=\beta$ as the sample size grows large) and when all variants are valid, any variation between the ${\hat{\beta}_{R}}_{j}$s should be due to sampling variation alone. However, when $\alpha_{j}$≠0 for some j, ${\hat{\beta}_{R}}_{j}=\beta+b_{j}$, where $b_{j}={\alpha_{j}}/{\beta_{Xj}}$ (i.e., a bias term). Therefore, the IVW estimate is only a consistent estimate for $\beta$ if these bias terms (a) sum to zero and (b) are independent in magnitude of the $\beta_{Xj}$ parameters (i.e., balanced pleiotropy under the InSIDE assumption[^1^](#_ENREF_1)). Assessing the heterogeneity between the ${\hat{\beta}_{R}}_{j}$s and the adoption of random effects models have therefore been proposed as strategies to detect horizontal pleiotropy.[^2^](#_ENREF_2)^,^[^3^](#_ENREF_3)

Two new methods for summary data MR that offer increased robustness to directional horizontal pleiotropy (i.e., bias terms do not sum to zero) have been proposed: MR-Egger regression[^1^](#_ENREF_1) and the weighted median.[^4^](#_ENREF_4) Briefly, MR-Egger regression fits an inverse-variance weighted linear regression of $\hat{\beta}_{Yj}$ against $\hat{\beta}_{Xj}$. Its intercept can be interpreted as a test of directional horizontal pleiotropy. It can provide a consistent estimate even when there is directional horizontal pleiotropy, as long as the InSIDE assumption holds. The weighted median estimate is the 50^th^ percentile of the inverse-variance weighted empirical distribution function of all ${\hat{\beta}_{R}}_{j}$s. It is a consistent estimate if at least 50% of the weight in the analysis stems from valid instruments.

**Analogy between the mode-based estimate (MBE) and the histogram**

An analogy can be drawn between the proposed MBE method and histograms. Consider a hypothetical bimodal variable $V$, which was simulated by concatenating two normally distributed variables: $V_{1}\sim N(0,1)$ and $V_{2}\sim N(5,25)$. In this example, the mode of $V$ would be zero, because the distribution with the smallest dispersion is centred at zero. We can use histograms to obtain a rough mode estimate of $V$ using the midpoint of the highest bar.

To make the histogram, it is necessary to define the break points of each bar, which here will be equally-spaced, starting from the minimum value of $V$ and ending at the maximum value of $V$. As shown in Supplementary Figure 1A, using six equally-spaced bars does not provide enough resolution to detect bimodality in $V$. As a result, the mode estimate is biased upwards. In panel B (20 equally-spaced bars), it can be clearly seen that $V$ is bimodal, and the mode estimate is less biased. However, when 100 equally-spaced bars are used (panel C), the data appears to be multimodal, because such spacing criteria was too stringent, so that random fluctuations under the same underlying distribution end up looking like being different distributions in the histogram. While this would not be a problem in infinite samples, it results in numerical instability of the mode estimate in finite samples. So, among panels A-C, the histogram in panel B provides the best bias-variance trade-off, and would be the best one to display the distribution of $V$.

As panels D-F illustrate, the proposed smoothed empirical density function approach is analogous to the histogram: the smoothed empirical density function is roughly similar to connecting the midpoints of each bar of the histogram (in other words, a “smoothed histogram”); the mode estimate based on the smoothed empirical density function is roughly similar to the mode estimate using the midpoint of the highest bar; and decreasing the tuning parameter $\varphi$ is analogous to increasing the number of equally-spaced bars in the histogram. Panels D-F also illustrate that the mode (as estimated by our proposed method) and the mean can be substantially different in case of bimodality, and our mode estimate being close to the centre of the main distribution.

**Summary data interpretation of the simulation model**

Although our underlying model is generated at the individual level, all of the methods we will investigate are applied directly to the set of $L$ SNP-exposure and SNP-outcome association estimates (written as $\hat{\beta}_{Xj}$ and $\hat{\beta}_{Yj}$ in models (1) and (2) described in the main text). These summary data parameters can be linked to the individual participant data parameters as follows. Firstly, let ${Z_{U}}_{ij}$, ${Z_{X}}_{ij}$ and ${Z_{Y}}_{ij}$ represent the $j$th component of ${Z_{U}}_{i}$, ${Z_{X}}_{i}$ and ${Z_{Y}}_{i}$, respectively. We then have that

$${\beta_{X}}_{j}G_{ij}=\gamma_{X}{Z_{X}}_{ij}+\gamma_{U}\theta_{X}{Z_{U}}_{ij}\text{ (11)}$$

$${\beta_{Y}}_{j}G_{ij}=\gamma_{Y}{Z_{Y}}_{ij}+\gamma_{U}\theta_{Y}{Z_{U}}_{ij}+\beta\left( \gamma_{X}{Z_{X}}_{ij}+\gamma_{U}\theta_{X}{Z_{U}}_{ij} \right),\text{ (12)}$$

which yields the ratio estimand

$${\beta_{R}}_{j}=\beta+\frac{\gamma_{Y}\frac{{\delta_{Y}}_{j}}{\sigma_{ZY}}+\gamma_{U}\theta_{Y}\frac{{\delta_{U}}_{j}}{\sigma_{ZU}}}{\gamma_{X}\frac{{\delta_{X}}_{j}}{\sigma_{ZX}}+\gamma_{U}\theta_{X}\frac{{\delta_{U}}_{j}}{\sigma_{ZU}}}=\beta+\frac{Total direct effect of G_{j}}{Instrument strength of G_{j}}=\beta+\frac{\alpha_{j}}{{\beta_{X}}_{j}}.\text{ (13)}$$

Variant$j$therefore gives a consistent estimate for $\beta$ only when the total direct effect of $G_{j}$ on $Y$=0. This is equivalent to $a_{j}$ in model (2). The IVW estimate is consistent if the total direct effect is zero for all variants and InSIDE holds. The weighted median is consistent if at least 50% of the information in the analysis stems from variants with a zero direct effect. MR-Egger regression can consistently estimate the causal effect if, across all variants, the total direct effects are independent of the instrument strengths (i.e., InSIDE holds). This is only true in general when horizontal pleiotropy does not affect $Y$ via a confounder ($\gamma_{U}$=0), so that the ratio estimand for variant $G_{j}$ is:

$${\beta_{R}}_{j}=\beta+\frac{\gamma_{Y}\frac{{\delta_{Y}}_{j}}{\sigma_{ZY}}}{\gamma_{X}\frac{{\delta_{X}}_{j}}{\sigma_{ZX}}}.\text{ (14)}$$

Finally, the MBE estimate is consistent under ZEMPA – i.e., when the most frequent total direct (or pleiotropic) effect is zero (in the case of the simple MBE).

**References**

1. Bowden J, Davey Smith G, Burgess S. Mendelian randomization with invalid instruments: effect estimation and bias detection through Egger regression. *Int J Epidemiol* 2015; 44(2):512-525.

2. Greco MF, Minelli C, Sheehan NA, Thompson JR. Detecting pleiotropy in Mendelian randomisation studies with summary data and a continuous outcome. *Stat Med* 2015; 34(21):2926-2940.

3. Bowden J, Del Greco M F, Minelli C, Davey Smith G, Sheehan NA, Thompson JR. A framework for the investigation of pleiotropy in two-sample summary data Mendelian randomization. *Stat Med* 2017; In press.

4. Bowden J, Davey Smith G, Haycock PC, Burgess S. Consistent Estimation in Mendelian Randomization with Some Invalid Instruments Using a Weighted Median Estimator. *Genet Epidemiol* 2016; 40(4):304-314.

**Software (R language and environment for statistical computing and graphics) code for implementing the MBE**

#BetaXG: vector of instrument-exposure regression coefficients

#BetaYG: vector of instrument-outcome regression coefficients

#seBetaXG: vector of instrument-exposure standard errors (SEs)

#seBetaYG: vector of instrument-outcome SEs

#phi: tunning parameter (e.g., 1=default bandwidth; 0.5=half of the default bandwidth)

#n_boot: number of bootstrap iterations

#alpha: alpha level of the confidence intervals (e.g., alpha=0.05 corresponds to 1-0.05=95% confidence intervals)

MBE <- function(BetaXG, BetaYG, seBetaXG, seBetaYG, phi=c(1,0.5,0.25), n_boot=1e4, alpha=0.05) {

#--------------------------------------#

#Function to compute the point estimate#

#--------------------------------------#

#BetaIV.in: ratio estimates

#seBetaIV.in: standard errors of ratio estimates

beta <- function(BetaIV.in, seBetaIV.in) {

#Bandwidth rule - modified Silverman's rule proposed by Bickel (2002)

s <- 0.9*(min(sd(BetaIV.in), mad(BetaIV.in)))/length(BetaIV.in)^(1/5)

#Standardised weights

weights <- seBetaIV.in^-2/sum(seBetaIV.in^-2)

beta <- NULL

for(cur_phi in phi) {

#Define the actual bandwidth

h <- s*cur_phi

#Compute the smoothed empirical density function

densityIV <- density(BetaIV.in, weights=weights, bw=h)

#Extract the point with the highest density as the point estimate

beta[length(beta)+1] <- densityIV$x[densityIV$y==max(densityIV$y)]

}

return(beta)

}

#------------------------------------------#

#Function to estimate SEs through bootstrap#

#------------------------------------------#

#BetaIV.in: ratio estimates

#seBetaIV.in: standard errors of ratio estimates

#beta_MBE.in: point causal effect estimates

boot <- function(BetaIV.in, seBetaIV.in, beta_MBE.in) {

#Set up a matrix to store the results from each bootstrap iteration

beta.boot <- matrix(nrow=n_boot, ncol=length(beta_MBE.in))

for(i in 1:n_boot) {

#Re-sample each ratio estimate using SEs derived not assuming NOME

BetaIV.boot <- rnorm(length(BetaIV.in), mean=BetaIV.in, sd=seBetaIV.in[,1])

#Re-sample each ratio estimate using SEs derived under NOME

BetaIV.boot_NOME <- rnorm(length(BetaIV.in), mean=BetaIV.in, sd=seBetaIV.in[,2])

#Simple MBE, not assuming NOME

beta.boot[i,1:length(phi)] <- beta(BetaIV.in=BetaIV.boot, seBetaIV.in=rep(1, length(BetaIV)))

#Weighted MBE, not assuming NOME

beta.boot[i,(length(phi)+1):(2*length(phi))] <- beta(BetaIV.in=BetaIV.boot, seBetaIV.in=seBetaIV.in[,1])

#Simple MBE, assuming NOME

beta.boot[i,(2*length(phi)+1):(3*length(phi))] <- beta(BetaIV.in=BetaIV.boot_NOME, seBetaIV.in=rep(1, length(BetaIV)))

#Weighted MBE, assuming NOME

beta.boot[i,(3*length(phi)+1):(4*length(phi))] <- beta(BetaIV.in=BetaIV.boot_NOME, seBetaIV.in=seBetaIV.in[,2])

}

return(beta.boot)

}

#Ratio estimates

BetaIV <- BetaYG/BetaXG

#SEs of ratio estimates

seBetaIV <- cbind(sqrt((seBetaYG^2)/(BetaXG^2) + ((BetaYG^2)*(seBetaXG^2))/(BetaXG^4)), #SEs NOT assuming NOME

seBetaYG/abs(BetaXG)) #SEs ASSUMING NOME

#Point causal effect estimate using the simple MBE

beta_SimpleMBE <- beta(BetaIV.in=BetaIV, seBetaIV.in=rep(1, length(BetaIV)))

#Point causal effect estimate using the weighted MBE (not asusming NOME)

beta_WeightedMBE <- beta(BetaIV.in=BetaIV, seBetaIV.in=seBetaIV[,1])

#Point causal effect estimate using the weighted MBE (asusming NOME)

beta_WeightedMBE_NOME <- beta(BetaIV.in=BetaIV, seBetaIV.in=seBetaIV[,2])

#Combine all point effect estimates in a single vector

beta_MBE <- rep(c(beta_SimpleMBE, beta_WeightedMBE,

beta_SimpleMBE, beta_WeightedMBE_NOME))

#Compute SEs, confidence intervals and P-value

beta_MBE.boot <- boot(BetaIV.in=BetaIV, seBetaIV.in=seBetaIV, beta_MBE.in=beta_MBE)

se_MBE <- apply(beta_MBE.boot, 2, mad)

CIlow_MBE <- beta_MBE-qnorm(1-alpha/2)*se_MBE

CIupp_MBE <- beta_MBE+qnorm(1-alpha/2)*se_MBE

P_MBE <- pt(abs(beta_MBE/se_MBE), df=length(BetaXG)-1, lower.tail=F)*2

#Vector to indicate the method referring to each row

Method <- rep(c('Simple', 'Weighted', 'Simple (NOME)', 'Weighted (NOME)'), each=length(phi))

#Return a data frame containing the results

Results <- data.frame(Method, phi, beta_MBE, se_MBE, CIlow_MBE, CIupp_MBE, P_MBE)

colnames(Results) <- c('Method', 'phi', 'Estimate', 'SE', 'CI_low', 'CI_upp', 'P')

return(Results)

}
